# The Combined Konno-Nicks Procedure for Severely Stenotic Aortic Root and Left Ventricular Outflow Tract

**DOI:** 10.1016/j.jaccas.2025.106544

**Published:** 2026-01-28

**Authors:** Masaru Matsuda, Shin Yajima, Daisuke Yoshioka, Kazuo Shimamura, Ai Kawamura, Takuji Kawamura, Yusuke Misumi, Shunsuke Saito, Shigeru Miyagawa

**Affiliations:** Department of Cardiovascular Surgery, University of Osaka, Suita, Japan

**Keywords:** aortic root enlargement, Konno procedure, Nicks procedure, prosthetic valve dysfunction, subvalvular stenosis

## Abstract

**Background:**

Patients with a small aortic root and subvalvular stenosis are at risk of prosthesis-patient mismatch, making annular and left ventricular outflow tract (LVOT) enlargement essential.

**Case Summary:**

We herein report the case of a 41-year-old woman with prosthetic valve dysfunction and severe LVOT obstruction due to pannus formation in whom a 12-mm sizer could not be passed. Redo aortic valve replacement with the combined Konno-Nicks procedure successfully enlarged the annulus and LVOT, enabling the implantation of a 4-size larger prosthesis based on intraoperative sizer measurements. Postoperative echocardiography and computed tomography confirmed effective valve enlargement and preserved valve function.

**Discussion:**

Combined anterior-posterior enlargement can be valuable for redo aortic valve replacement in patients with a small annulus and subvalvular stenosis to prevent prosthesis-patient mismatch.

**Take-Home Message:**

The combined Konno-Nicks procedure can effectively relieve prosthetic valve dysfunction with severe subvalvular stenosis by enabling larger prosthesis implantation.

## Visual Summary

**Figure:**
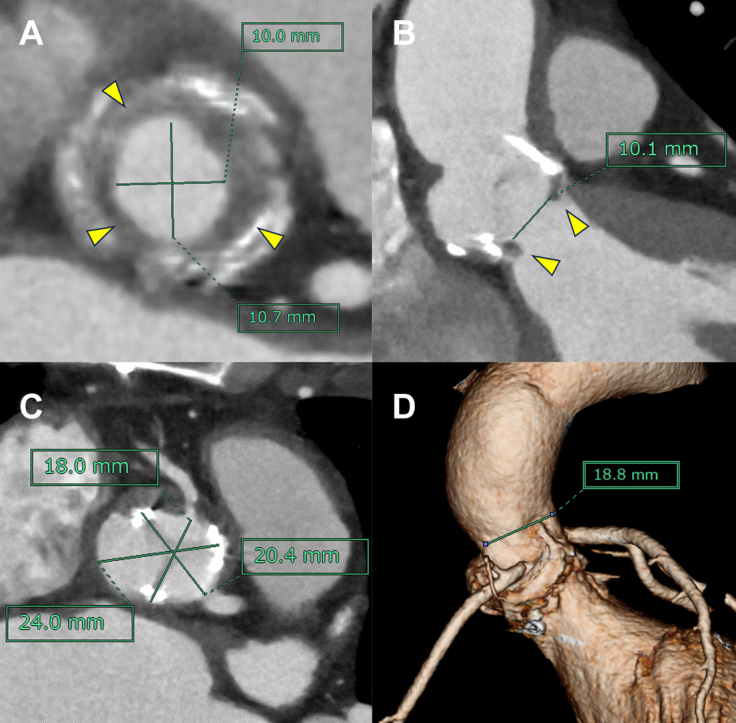

## History of Presentation

At 33 years of age, the patient underwent aortic valve replacement (AVR) with a 19-mm Carpentier-Edwards Perimount bioprosthesis (Edwards Lifesciences) and the Nicks procedure for rheumatic aortic stenosis and a small aortic root. A bioprosthetic valve was selected to allow for future pregnancy. The mean transvalvular pressure gradient (PG) after the initial surgery was 17 mm Hg. She was followed up annually with echocardiography, and the mean PG remained at approximately 14 mm Hg for 2 years, until she was lost to follow-up. At 41 years of age, approximately 3 months before the current admission, she developed exertional chest pain and dyspnea and returned to our hospital. At admission, her NYHA functional class was class II; her height, weight, and body surface area were 150 cm, 46 kg, and 1.38 m^2^, respectively.

## Past Medical History

The patient had no medical history of chronic kidney disease, diabetes mellitus, or hypertension.

## Differential Diagnosis

Differential diagnoses included prosthetic valve stenosis and coronary artery stenosis.

## Investigations

**Figure 1:**
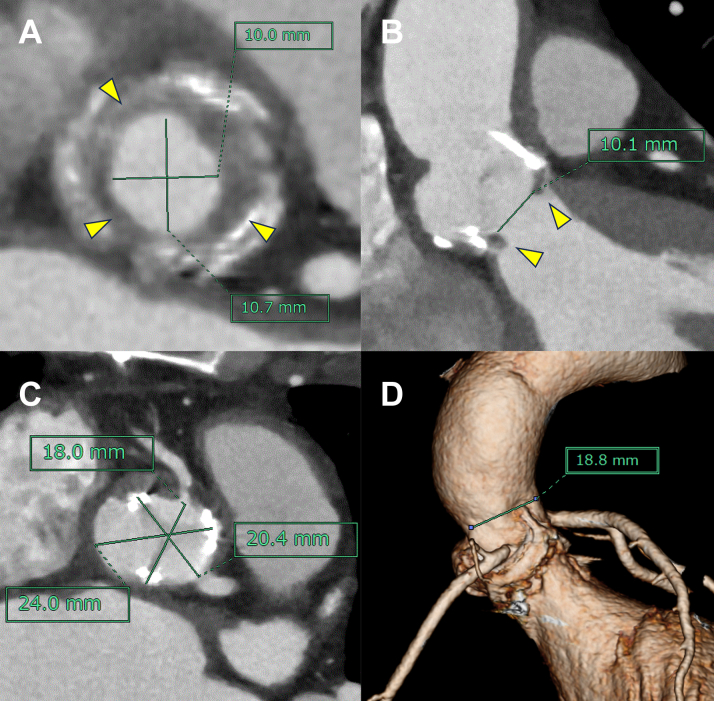
Preoperative Computed Tomography Subvalvular stenosis is shown in the short-axis (A) and long-axis (B) views. The yellow arrowhead indicates pannus. The small root and left ventricular outflow tract are shown in the short-axis view (C) and 3-dimensional reconstruction (D).

Preoperative transthoracic echocardiography revealed prosthetic valve dysfunction (PVD) with an effective orifice area of 0.92 cm^2^, a mean PG of 33 mm Hg, and a peak PG of 59 mm Hg, as well as a peak velocity across the aortic valve of 3.8 m/s. In addition, left ventricular outflow tract (LVOT) stenosis was noted with a peak velocity of 1.9 m/s. The left ventricular ejection fraction (LVEF) was 76%. The *E*/*A* ratio was 1.42, and the *E*/*e*ʹ ratio was 18. Contrast-enhanced computed tomography (CT) demonstrated mild structural valve degeneration and severe subvalvular stenosis with pannus formation, with the narrowest diameter being 10.1 mm (Figures 1A and 1B). The LVOT measured 17.5 mm and 11.3 mm along the long and short axes, respectively. The diameters of the sinuses of Valsalva were 18.0 mm, 20.4 mm, and 24.0 mm (Figure 1C). The sinotubular junction diameter was 18.8 mm (Figure 1D). No coronary stenosis was observed. However, the right coronary artery (RCA) ostium was approximately 3 mm from the stent post at the commissure between the right and left coronary cusps of the previous valve.

## Management

Redo AVR with root enlargement was planned owing to symptomatic PVD with a small aortic root and subvalvular stenosis. Because the RCA ostium was close to the Konno incision line, prophylactic coronary artery bypass grafting (CABG) to the RCA was scheduled.

**Figure 2:**
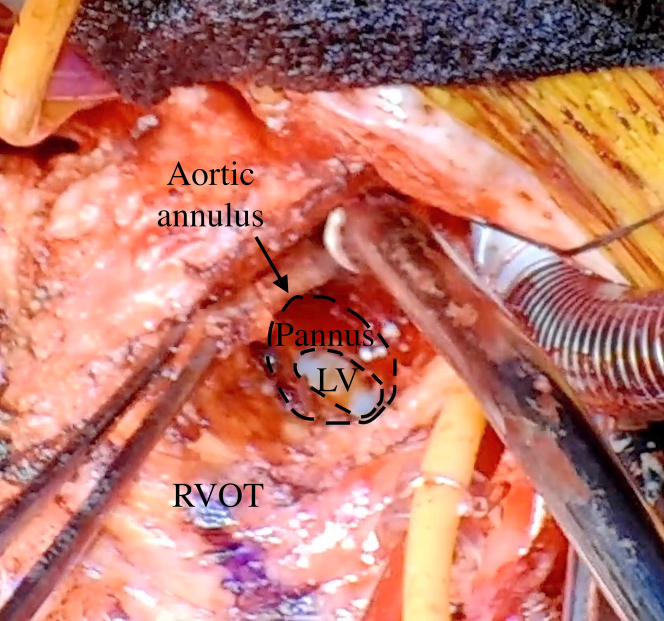
Intraoperative Findings The image shows a severely stenotic aortic valve annulus extensively covered with pannus. The arrow indicates the aortic annulus. LV = left ventricle; RVOT = right ventricle outflow tract.

**Figure 3:**
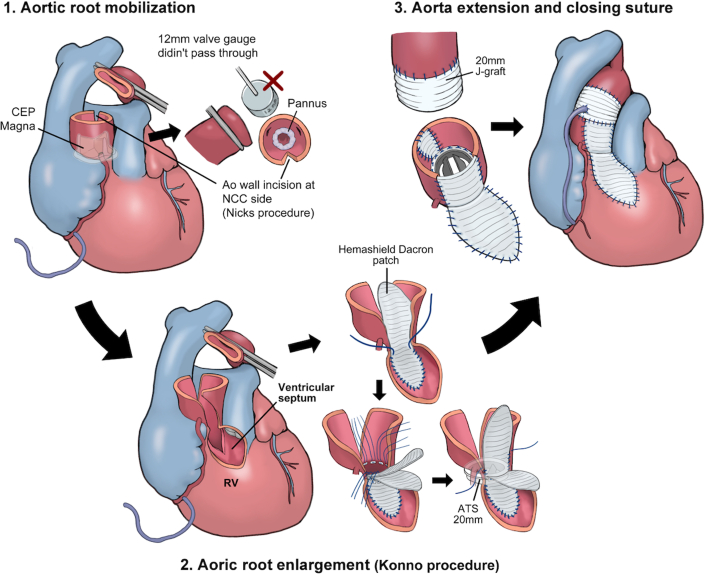
Schematic Illustration of the Procedure The aortic annulus was severely stenotic, preventing passage of a 12-mm sizer. Aortic root enlargement using the Konno-Nicks procedure enabled the implantation of a 20-mm aortic valve. ATS = ATS AP360 mechanical valve (Medtronic); CEP = Carpentier-Edwards Perimount Magna Ease bioprosthesis (Edwards Lifesciences); NCC = noncoronary cusp; RV = right ventricle.

After cardiopulmonary bypass establishment and before aortic cross-clamping, distal anastomosis of the saphenous vein graft (SVG) was performed to the midportion of the RCA in an end-to-side fashion. Afterward, the aortic valve procedure was performed. The explanted prosthesis revealed a severely narrowed annulus with pannus overgrowth (Figure 2). Even after pannus removal, a 12-mm sizer could not be passed through the annulus. A posterior aortic incision was extended toward the noncoronary cusp, and an anterior incision into the right ventricular free wall was made 2 cm into the interventricular septum, enabling the passage of a 19-mm sizer. The root was enlarged using 2 Hemashield Dacron patches (Boston Scientific). The inner patch closes the intraventricular defect, whereas the outer patch reinforces the right ventricle using pledgeted sutures. A 20-mm ATS AP360 prosthesis (Medtronic) was implanted using 11 pairs of pledgeted sutures. The posterior incision was augmented using a diamond-shaped Hemashield patch. Severe adhesions required an interposed 20-mm J-graft vascular prosthesis (Japan Lifeline) for proximal-distal aortic reconstruction. The right ventricle was reinforced using an outer patch. Subsequently, proximal anastomosis of the CABG was performed to the interposed ascending aortic graft (Figure 3). Transit-time flow measurements demonstrated a mean flow of 49 mL/min and a pulsatility index of 1.0.

## Outcome and Follow-Up

**Figure 4:**
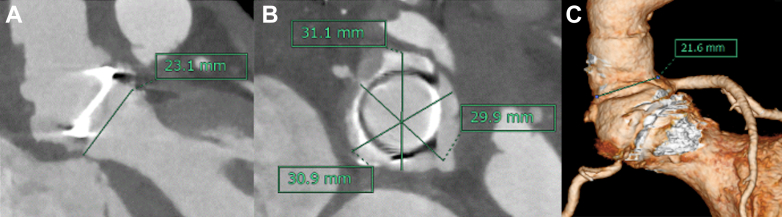
Postoperative Computed Tomography Postoperative computed tomography in the short-axis view (A), long-axis view (B), and 3-dimensional reconstruction (C) demonstrate successful enlargement of the aortic root and left ventricular outflow tract.

The total operative time was 7 hours and 42 minutes, with cardiopulmonary bypass and aortic cross-clamp times of 313 minutes and 219 minutes, respectively. Postoperative echocardiography revealed a mean transvalvular PG of 6 mm Hg, a peak PG of 13 mm Hg, and a peak velocity of 1.8 m/s, with no residual LVOT stenosis. The LVEF was 78%. No aortic regurgitation and only trivial mitral and tricuspid regurgitation were observed. The effective orifice area could not be calculated because of limited echocardiographic visualization due to prosthetic valve artifacts and early postoperative mediastinal changes. Postoperative CT confirmed subvalvular widening to 23.1 mm (Figure 4A). The diameters of the sinuses of Valsalva were 29.9 mm, 31.1 mm, and 30.9 mm (Figure 4B). The sinotubular junction diameter was 21.6 mm (Figure 4C). These findings indicated successful root and LVOT enlargement. CT also showed a patent SVG. The patient recovered uneventfully and was discharged without any symptoms on postoperative day 29. At the 1-year postoperative follow-up, the patient remained asymptomatic with no adverse cardiac events. Transthoracic echocardiography revealed normal left ventricular contraction without asynergy and an LVEF of 70%. The prosthetic aortic valve showed normal function, with a peak velocity of 2.0 m/s, a mean PG of 10 mm Hg, and an effective orifice area of 2.0 cm^2^. No LVOT stenosis was observed. No perivalvular or transvalvular leakage was detected.

## Discussion

In the current era of valve-in-valve transcatheter AVR and lifetime management of aortic valve disease, patients with a small aortic annulus are at increased risk of developing prosthesis-patient mismatch (PPM), which may negatively affect long-term outcomes. PPM is associated with higher mortality after surgical AVR,^1^ transcatheter AVR,^2^ and valve-in-valve transcatheter AVR.^3^ Therefore, aortic annulus and root enlargement at the initial operation is particularly important, because it facilitates implantation of an adequately sized prosthesis and preserves future treatment options by permitting the placement of a larger valve during subsequent valve-in-valve procedures. Although aortic annulus and root enlargement serves as an effective adjunct to surgical AVR, the benefit of reducing PPM must be balanced against the potentially high risk of perioperative mortality that is associated with these procedures.^4^

In the present case, the initially small aortic root was further narrowed because of adhesions and wall thickening after the prior surgery. Even after pannus removal, the annular and LVOT narrowing persisted. The Konno procedure was selected over other techniques for several reasons. For instance, although the Manouguian and Y-incision techniques permit upsizing by ≥2 valve sizes, each has limitations. Namely, the Manouguian approach involves the mitral valve, risking regurgitation or replacement, whereas the Y-incision avoids mitral involvement but provides only horizontal augmentation, risking valve-LVOT mismatch and residual PG.^5^ In contrast, the Konno procedure, first described by Konno et al,^6^ enlarges the annulus anteriorly through a septal incision, effectively relieves LVOT obstruction, and allows for large prosthetic valve implantation. Nevertheless, it is associated with risks including atrioventricular block or residual ventricular shunt.

Few reports using the combined Konno-Nicks procedure have been published. In 1991, Chou et al^7^ reported a case in which they used the combined Konno-Nicks^8^ procedure. Another related case, reported by Matsuzaki et al^9^ in 2015, described a Konno procedure performed during redo AVR after a previous Nicks procedure. Both reports are available only as abstracts without postoperative details. Because reports combining these 2 procedures are scarce, outcome data are lacking.

Regarding the Konno procedure, major complications include reoperation (up to 39% at 10 years), prosthetic valve thrombosis, pannus formation, infective endocarditis, thromboembolism, and early mortality (up to 8%), with a 10-year survival rate of 86%. For the Nicks procedure, data are limited, but the main reported complications include PVD, pannus-related obstruction, and reoperation.

Another approach, the Yamaguchi procedure,^10^ achieves bidirectional enlargement of the aortic root; however, the incision is confined to the aortic annulus and does not extend into the right ventricle or interventricular septum. In contrast, the combined Konno-Nicks procedure allows for more extensive annular enlargement and effective relief of LVOT obstruction. Nonetheless, the potential disadvantages of this approach include the risk of complete atrioventricular block and residual ventricular septal defects due to the septal incision.

The risk of right coronary ostial injury during the Konno incision was substantial. Preoperative CT showed that the right coronary ostium was approximately 3 mm from the stent post at the commissure of the right and left coronary cusps of the previous valve. Given the narrow margins for incision and patching, the potential for ostial stenosis was high. Postoperative CT revealed new narrowing of the right coronary ostium that was not present preoperatively, suggesting that prophylactic CABG to the RCA likely prevented ischemia.

In the absence of coronary stenosis, competitive flow between the native RCA and the SVG can lead to early graft occlusion. However, bypass was performed as a prophylactic measure; therefore, graft occlusion would not be clinically significant, because it likely reflects adequate native coronary flow. Coronary button reimplantation may be an alternative approach. Accordingly, prophylactic CABG to the RCA was considered the most reasonable and safe option to prevent myocardial ischemia due to right coronary ostial narrowing.

## Conclusions

The presented case demonstrates that the combined Konno-Nicks procedure is feasible for redo AVR in patients with a small aortic root and subvalvular stenosis, allowing larger prosthesis implantation and effective LVOT obstruction relief.

### Data Availability Statement

All data generated in this study are included in this paper.

**Take-Home Messages**

• The combined Konno-Nicks procedure can effectively enlarge the aortic annulus and left ventricular outflow tract in redo aortic valve replacement in patients with a small aortic root and subvalvular stenosis.
• This approach enables the implantation of a larger prosthesis and provides an important surgical option for preventing prosthesis-patient mismatch.

## Funding Support and Author Disclosures

The authors have reported that they have no relationships relevant to the contents of this paper to disclose.
